# Isolated Fallopian Tube Torsion in a 14-Year-Old Girl: A Case Report and Review of the Literature

**DOI:** 10.7759/cureus.75853

**Published:** 2024-12-17

**Authors:** Masayoshi Nagahama, Erika Tomoyose, Yasuji Yoshikawa, Midori Tokashiki, Akira Hokama

**Affiliations:** 1 Department of Surgery, Naha City Hospital, Naha, JPN; 2 Department of Obstetrics and Gynecology, Naha City Hospital, Naha, JPN; 3 Department of Obstetrics and Gynecology, Graduate School of Medicine, University of the Ryukyus, Nishihara, JPN; 4 Department of Pathology, Naha City Hospital, Naha, JPN; 5 Department of Medical Checkup, Naha City Hospital, Naha, JPN

**Keywords:** acute abdomen in children, computed tomography abdomen, emergency medicine imaging, isolated fallopian tube torsion, laparoscopic gynaecological surgery

## Abstract

Isolated fallopian tube torsion (IFTT) is a rare cause of surgical emergency and is difficult to diagnose. We present a case of IFTT in a 14-year-old girl who presented with an acute abdomen. Based on the clinical and computed tomographic findings, an initial diagnosis of ovarian torsion was considered. However, an emergency laparoscopic surgery revealed a left necrotic hemorrhagic tubal torsion. Subsequently, a left salpingectomy was performed with the preservation of both ovaries. Histological examination confirmed the diagnosis of IFTT with hydrosalpinx. This case highlights the diagnostic challenges associated with IFTT and emphasizes the important role of early surgical intervention in preventing morbidity and preserving ovaries. A brief review of the literature on IFTT in terms of its diagnostic and therapeutic approaches is discussed.

## Introduction

Isolated fallopian tube torsion (IFTT) is defined as a torsion of the fallopian tube without concomitant ovarian torsion. IFTT is a rare cause of gynecological acute abdomen, with an estimated prevalence of one in 1.5 million women [1]. IFTT occurs primarily in women of reproductive age and is very rare in the pediatric age group. The clinical features are nonspecific, leading to delayed diagnosis. Herein, we report a case of IFTT in a 14-year-old girl, who was preoperatively diagnosed with ovarian torsion.

## Case presentation

**Figure 1:**
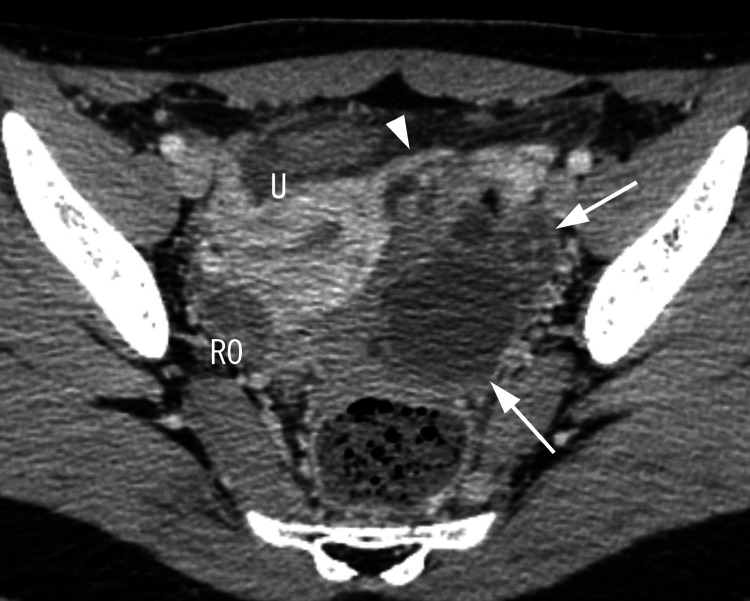
An axial pelvic CT image showing multiple cystic lesions (arrows) in the left adnexa consistent with ovarian torsion. The isthmus of the left fallopian tube was also observed (arrowhead) U: uterus; RO: right ovary.

A 14-year-old female with no past medical history presented to the emergency department with severe lower abdominal pain since the previous day. Her menstrual cycles were regular. On examination, the vital signs were as follows: a pulse rate of 91 beats per minute, blood pressure of 147/84 mmHg, respiratory rate of 16 breaths per minute, and a temperature of 36.6°C. There was a tenderness with a rebound at the right lower quadrant of her abdomen. Laboratory examination showed a white blood cell count of 10.6×10^3^/µL (reference range 3.3-8.6 ×10^3^/μL) and hemoglobin of 13.3 g/dL (reference range 11.5-15.0 g/dL). The urine pregnancy test was negative. A computed tomography (CT) scan was performed because of the clinical suspicion of acute abdomen. The CT showed multiple cystic lesions in the left adnexa consistent with ovarian torsion (Figure 1).

**Figure 2:**
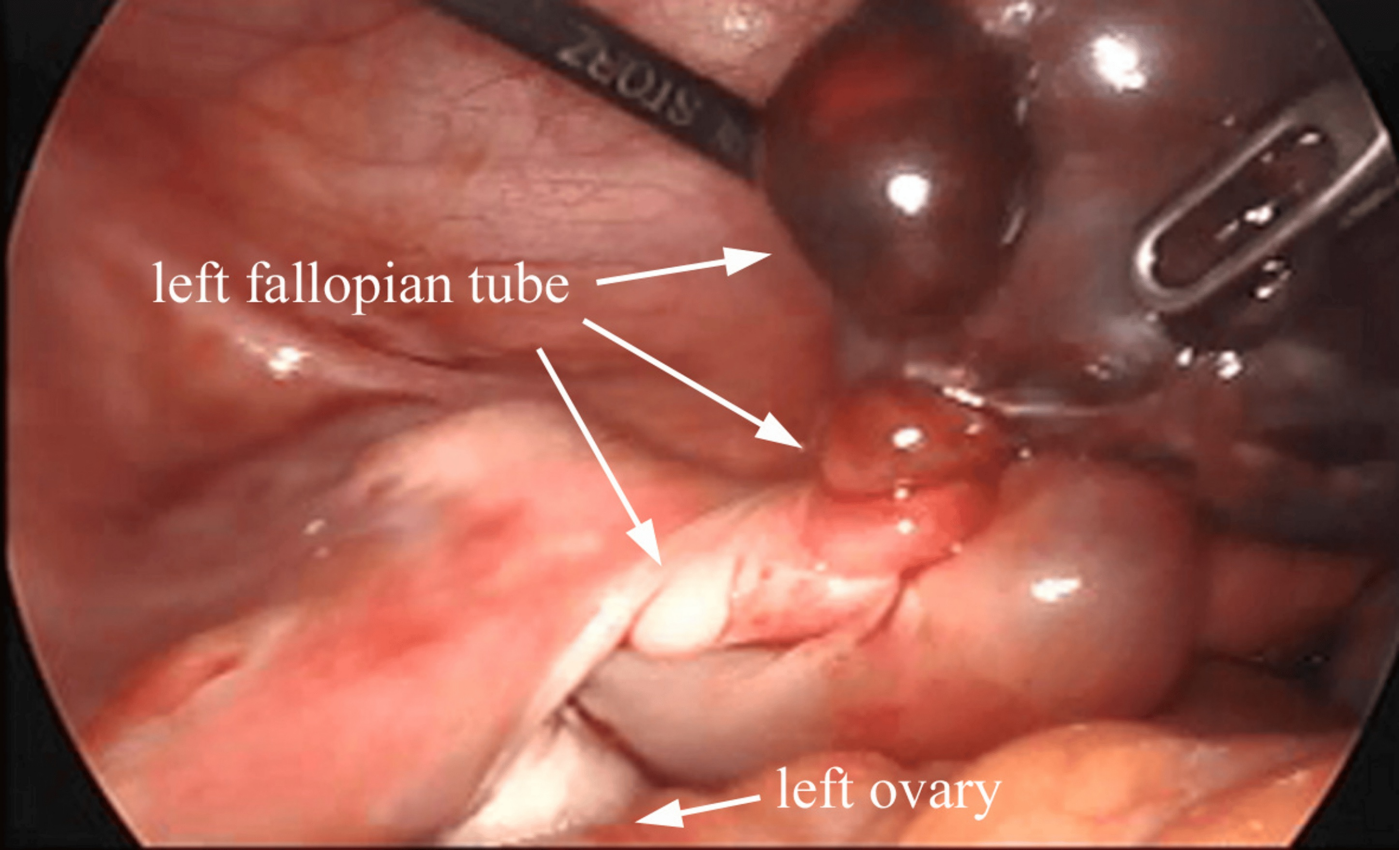
Laparoscopic view The central portion of the left fallopian tube was twisted multiple times around its axis, and the distal portion appeared enlarged and dark red from the torsion.

She underwent an emergency laparoscopy. On laparoscopy, the central portion of the left fallopian tube appeared twisted multiple times around its axis, and the distal portion was enlarged and dark red from the torsion (Figure 2).

**Figure 3:**
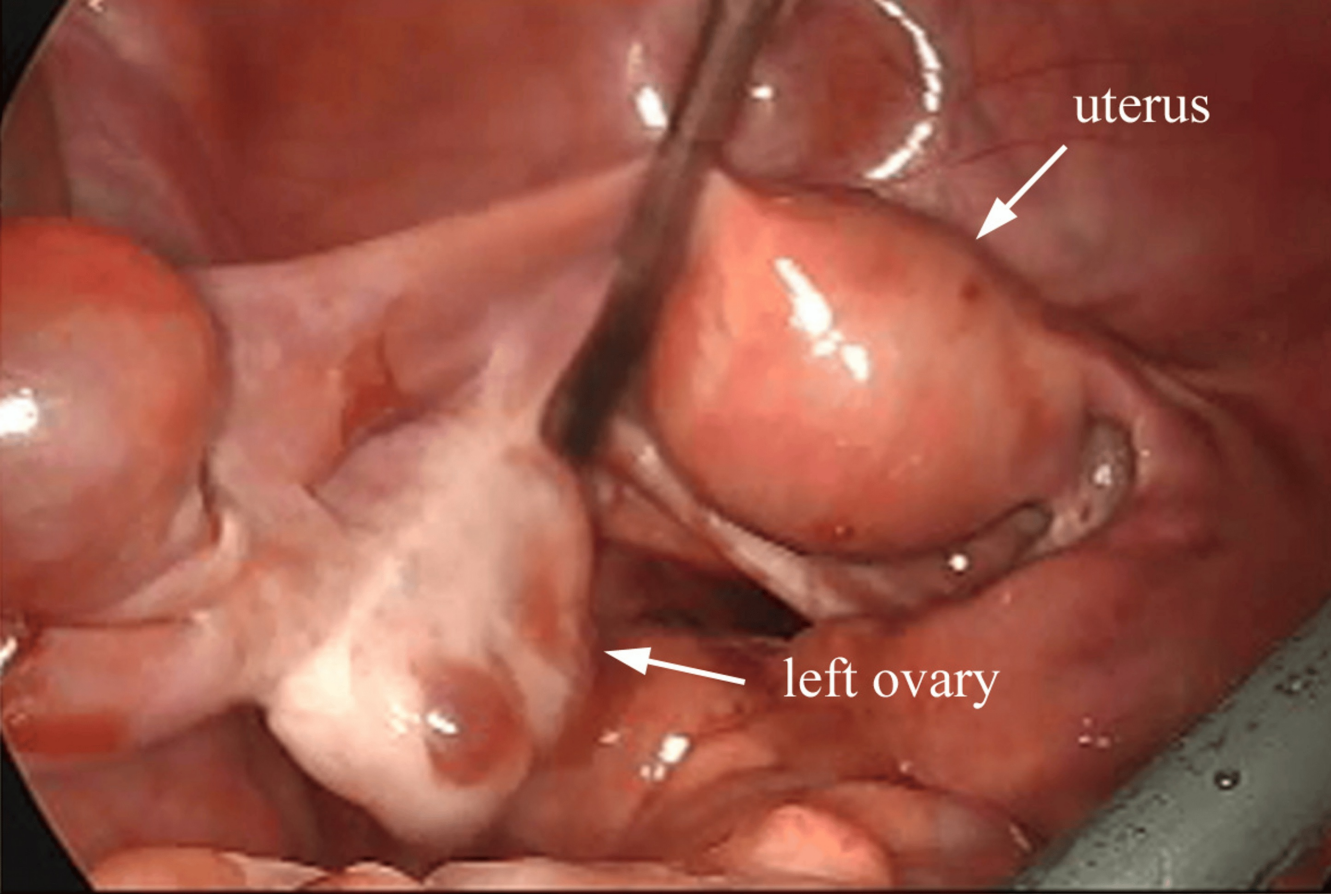
The left ovary appeared normal without torsion

The left ovary appeared normal without torsion (Figure 3).

**Figure 4:**
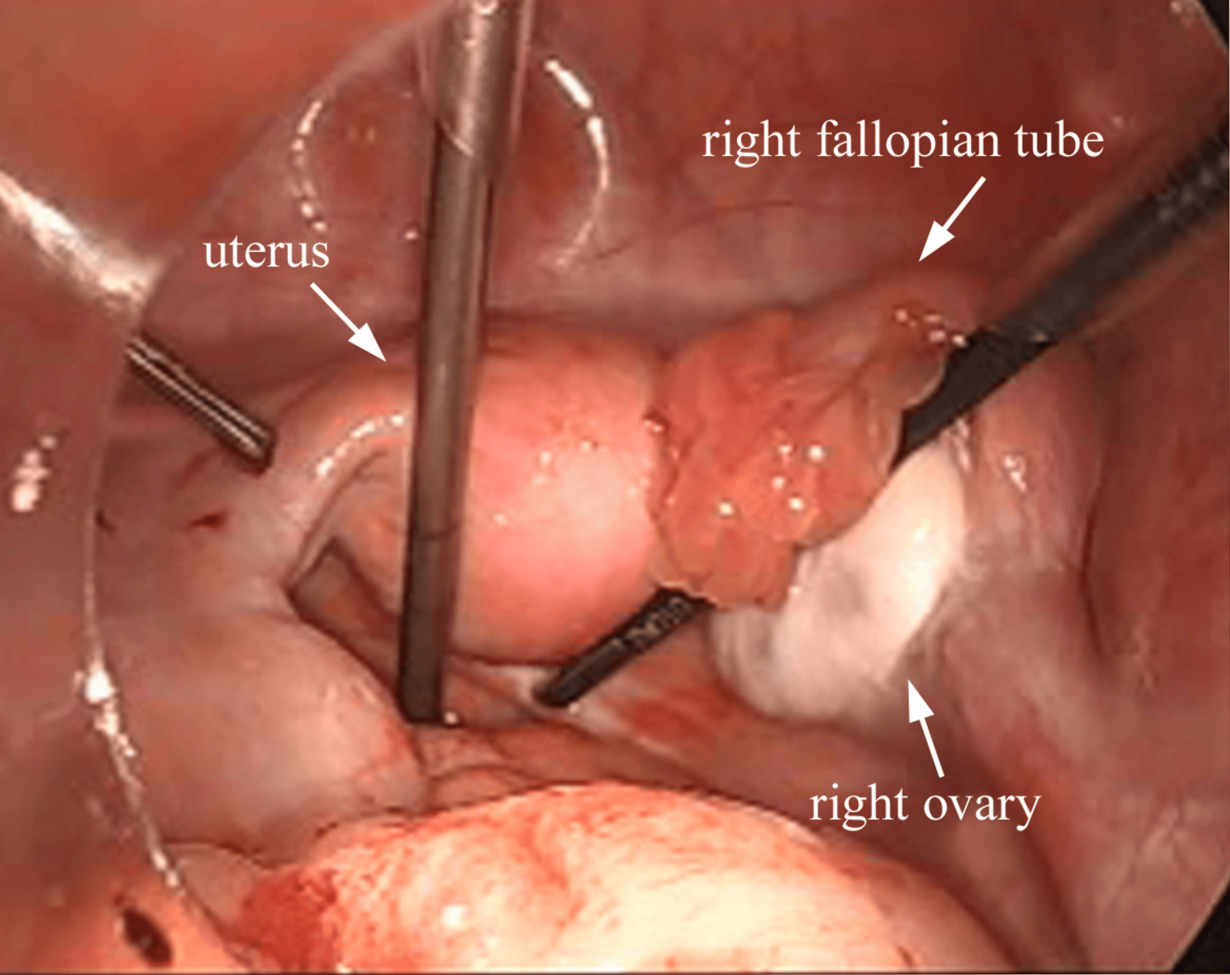
The uterus and the right adnexa were normal

The uterus and the right adnexa were also normal (Figure 4). There were no paraovarian cysts.

**Figure 5:**
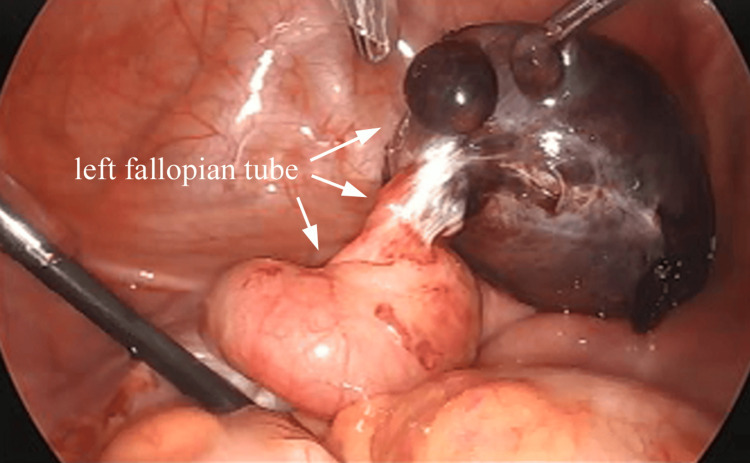
The left fallopian tube remained necrotic in appearance after detorsion

Detorsion of the left fallopian tube was performed but it remained necrotic (Figure 5) and a left salpingectomy was performed.

**Figure 6:**
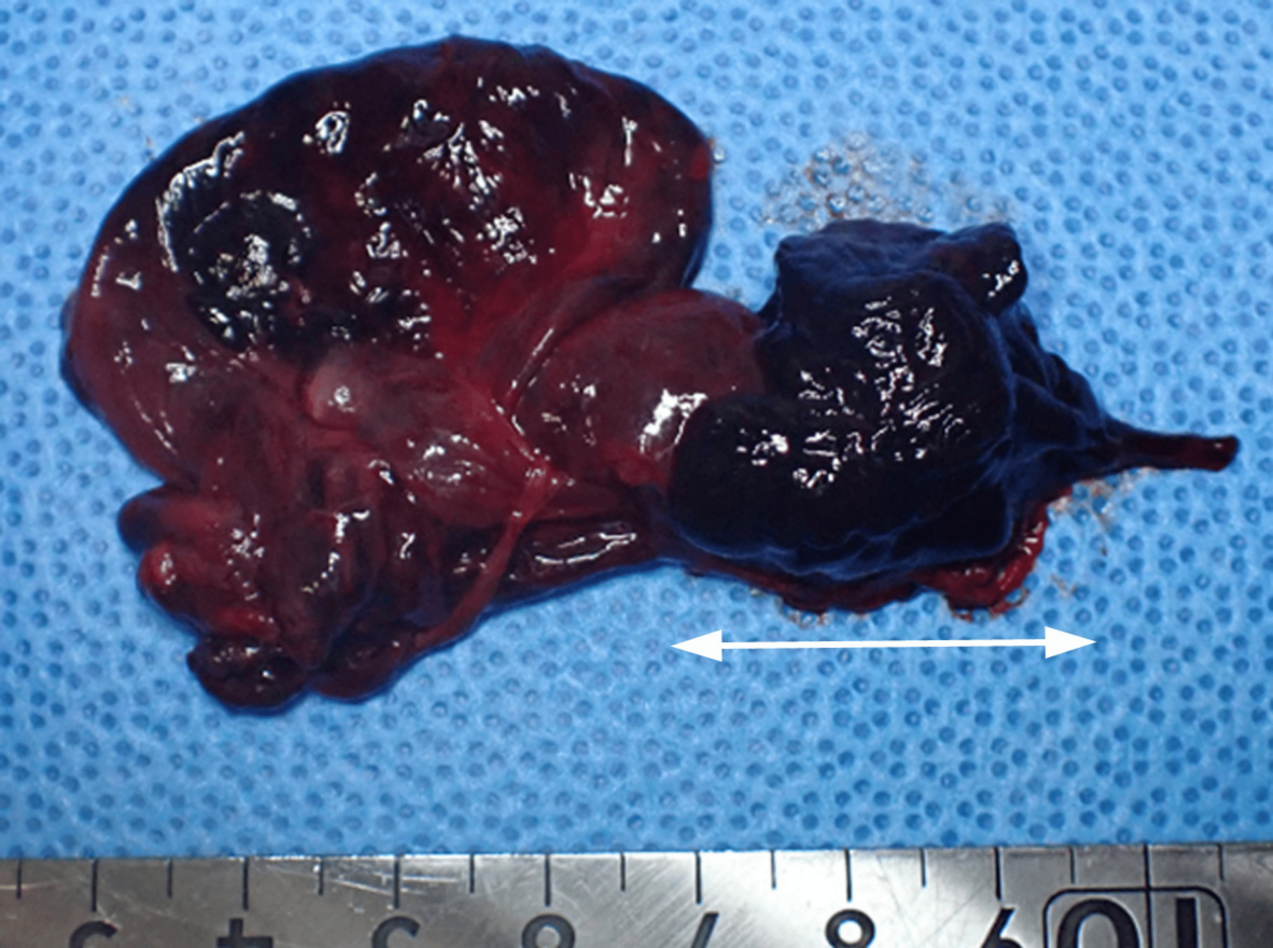
The gross view of the resected fallopian tube with the double-headed arrow showing the hemorrhagic hydrosalpinx

Histological examination confirmed hemorrhagic hydrosalpinx without evidence of malignancy or endometriosis (Figure 6).

The postoperative course was uneventful and the patient has remained asymptomatic for two years after the procedure.

## Discussion

IFTT is a rare form of adnexal torsion (AT) that is difficult to diagnose. The differential diagnoses in the pediatric age group includes ovarian torsion, ectopic pregnancy, pelvic inflammatory disease, acute appendicitis, and urinary tract disease. Several intrinsic and extrinsic factors can predispose a patient to tubal torsion [2]. Intrinsic factors include congenital tubal anomalies and acquired conditions such as an excessive length of the mesosalpinx, hydrosalpinx, hematosalpinx, cysts, neoplasms, and pelvic inflammatory disease, whereas, extrinsic factors include adnexal mass, adhesions, and accidental trauma [2]. In our case, the most likely factor is hydrosalpinx. The most common presenting complaint is acute onset of lower abdominal pain, which may be accompanied by nausea, vomiting, dysuria, and diarrhea [1,3]. These accompanying symptoms were not compatible with our case.

Although ultrasound is the first-line imaging modality, CT provides more important diagnostic information in the emergency setting [4,5]. A recent study identified two patterns of IFTT on CT [6]. The first was a thin-walled hydrosalpinx, U- or C-shaped, with a mean diameter of 3 cm. The second pattern was an extra-ovarian cyst adjacent to a soft tissue mass that contained the tortuous tube and vessels. In both patterns, the ipsilateral ovary was normal in size. Although the CT finding of our case was considered as the second pattern in the retrospective evaluation, the identification of extra- or intra-ovarian cysts was difficult among multiple adnexal cysts. This led to the preoperative diagnosis of ovarian torsion, which was later found to be IFTT at laparoscopy.

IFTT should be treated with immediate surgery. If the fallopian tube is not completely twisted and/or has not lasted long, it can be salvaged with a detorsion procedure. However, if the tube is necrotic, salpingectomy is the only option [7]. According to a study of AT in the pediatric and adolescent population, higher rates of oophorectomy and/or salpingectomy were associated with a longer duration of abdominal pain [8]. It is imperative to consider immediate imaging examination for the fertility prognosis when girls present with abdominal pain, nausea, and vomiting.

## Conclusions

IFTT is a rare cause of acute abdomen in pediatric girls. Its clinical features are nonspecific, leading to delayed diagnosis. In this case, the initial CT evaluation suggested ovarian torsion and subsequent laparoscopic surgery confirmed the diagnosis of IFTT and effective removal of the necrotic fallopian tube. This resulted in the preservation of the ipsilateral ovary. This case highlights the clinical recognition of IFTT and the importance of prompt surgical intervention for the fertility prognosis. 
